# Rheumatoid Arthritis in Minorities

**DOI:** 10.1155/2013/256493

**Published:** 2013-05-14

**Authors:** Juan-Manuel Anaya, Adriana Rojas-Villarraga, Rubén Darío Mantilla, Claudio Galarza-Maldonado

**Affiliations:** ^1^Center for Autoimmune Diseases Research (CREA), School of Medicine and Health Sciences, Universidad del Rosario, Carrera 24 No. 63-C-69, 11001000 Bogotá, Colombia; ^2^Rheumatology Unit, Riesgo de Fractura S. A.-CAYRE I.P.S., 11001000 Bogotá, Colombia; ^3^Rheumatic and Autoimmune Diseases Unit (UNERA), Mount Sinai Hospital, Miguel Cordero 6-111 y Avenida Solano, EC010150 Cuenca, Ecuador

Latin America and the Caribbean (LAC) is a rapidly growing region with almost 600 million inhabitants composed of Mexico, Central and South America, and the islands of the Caribbean [1, 2]. The Americas were first inhabited by people crossing the Bering Land Bridge from northeast Asia into Alaska well over 10,000 years ago. Native Americans descend from at least three streams of Asian gene flow [3]. Europeans arrived after 1492 following Christopher Columbus's voyages. African people were captured and taken to America by the transatlantic slave trade from the 16th to the 19th centuries. Hence, the population of LAC comprises a variety of ancestries, ethnic groups, and races, making the region one of the most diverse in the world. The specific composition varies from country to country: many have a predominance of European-Native American, or Mestizo, population; in others, Native Americans are a majority; some are dominated by inhabitants of European ancestry; some countries' populations are primarily Mulatto [4]. To a less extent, Black, Asian, and Zambo (mixed Black and Native American) are also identified regularly [4]. Noteworthy, ethnic self-identification is culturally and biologically complex and is not correlated with self-reported ancestry which should be no longer evaluated by questionnaire but rather by the use of ancestry informative markers (AIMs) at the molecular level [5].

The term majority refers to a group that controls economic, political, and social resources regardless of the population size. In this sense, LAC still meets most of the Feagin defining features of minority, including suffering discrimination and subordination, physical or cultural traits that set them apart, and a shared sense of collective identity and common burdens as well as socially shared rules [6]. LAC remains one of the world's most unequal regions [7]. Enormous cultural differences in health perceptions in LAC exist which correlate with individuals' economic and health conditions [8]. Lower-income groups recognize more health problems but are less tolerant to some of them than the rich [8].

There is an increased prevalence of chronic diseases in LAC which has been attributed to diverse causes, including ancestry, socioeconomic status (SES), the ageing of the population, and lifestyle factors such as smoking, physical inactivity, and excess of alcohol intake [8, 9]. Higher SES has been characterized by lower levels of Native American ancestry [9]. The prevalence of some autoimmune rheumatic diseases, including rheumatoid arthritis (RA), is higher than expected among some Amerindian groups highlighting ancestry as a factor influencing the risk of acquiring autoimmune diseases [10, 11]. 

In spite of setbacks, LAC is making important progress in research. The “Grupo Latino Americano de Estudio de Artritis Reumatoide (GLADAR)” is an example among several others (http://www.gladar.org/). From 2000 to 2010, LAC has seen a high growth of more than 9% per year in scholarly output, resulting in a nearly 70% increase in its share of world papers over the same period, to reach just under 4.4% of the world's annual output of scholarly papers in 2010 [12]. Latin American research is growing fast and becoming more visible on a global scale. And this is not the only bibliometrically-observed improvement to LAC's scholarly output over the last few years. LAC's relative citation impact, albeit still under world average, has been improving by 1.6% per year from 2000 to 2010 and from about 70% of world average in 2000 to more than four-fifths in 2010 [12]. Improving research and human resources capacity in the region will require increasing research partnerships within and outside the region, between rich and poor countries, promoting collaborations between LAC research institutions and universities to boost postgraduate programs, and aligning research investments and outputs with the current burden of disease [7].

This issue of *Arthritis* offers five papers from LAC. The effect of illness on workers participation and productivity, more than any other consequence of disease, is important to a wide range of stakeholders both within and outside the healthcare sector. R. C. del Moral and colleagues from Argentina report how patients with RA with higher disease severity show higher work productivity compromise.

Three reports from the Center for Autoimmune Diseases Research (CREA) in Colombia are presented. J. C. Roldan et al. evaluated the global prevalence of autoimmune thyroid disease (AITD) in RA, stressing that AITD should be systematically assessed since it is a risk factor for developing diabetes and cardiovascular disease (CVD) in RA. A systematic literature review in Latin America on CVD in RA, led by J. C. Sarmiento-Monroy, indicates a high prevalence of CVD in LA patients (35.3%). Main nontraditional risk factors associated to CVD in this population are HLA-DRB1 shared epitope alleles, rheumatoid factor, markers of chronic inflammation, long duration of RA, steroids, familial autoimmunity, and thrombogenic factors. Authors propose to evaluate cardiovascular risk factors comprehensively in the Latin RA patient and to generate specific public health policies in order to diminish morbimortality rates. J. Amaya-Amaya and colleagues report the usefulness of patients-reported outcomes (PROs) in RA focus group. Authors evaluated 135 patients with RA during two different sessions of focus group interviews. Agreement was found between objective measurements assessed by the physician and subjective assessments done by the patients regardless of gender, educational level, and duration of disease. Application of PROs in daily routine offers enormous benefits with patients' adherence to treatment and cost reductions as the most important. 

Finally, an elegant work from A. C. Machado-Díaz and colleagues from Cuba shows an increase of proinflammatory soluble interleukin-15 receptor alpha (IL-15R*α*) in patients with RA as compared with osteoarthritic patients. In addition, their results evidence the presence of IL-15R*α* in synovial fluids and suggest that its pro-inflammatory effect could be related to the induction of IL-6. 

We hope readers of *Arthritis* will enjoy this special issue and be aware of the importance and promises to investigate factors influencing health in minorities. As García Marquez said “solidarity with our dreams will not make us feel less alone, as long as it is not translated into concrete acts of legitimate support for all the peoples that assume the illusion of having a life of their own in the distribution of the world” [13].

*Juan-Manuel Anaya* *Adriana Rojas-Villarraga* *Rubén Darío Mantilla* *Claudio Galarza-Maldonado*
